# Severe and Atypical Presentation of Takotsubo Cardiomyopathy in a Pediatric Patient after a Serious Crash Injury—Case Report and Literature Review

**DOI:** 10.3390/pediatric15030036

**Published:** 2023-06-30

**Authors:** Christos Tsitsipanis, Marianna Miliaraki, Maria Michailou, Elisavet Geromarkaki, Anna-Maria Spanaki, Vasilia Nyktari, Andreas Yannopoulos, Nikolaos Moustakis, Stavroula Ilia

**Affiliations:** 1 Neurosurgery Department, University Hospital of Heraklion, 70013 Heraklion, Greece; 2 Pediatric Intensive Care Unit, University Hospital of Heraklion, 70013 Heraklion, Greece; 3 Pediatric Department, University Hospital of Heraklion, 70013 Heraklion, Greece; 4 Anaesthesiology Department, University Hospital of Heraklion, 70013 Heraklion, Greece

**Keywords:** takotsubo cardiomyopathy, traumatic brain injury, epidural hematoma

## Abstract

Takotsubo cardiomyopathy is an uncommon clinical entity in children, resulting in severe but sometimes reversible systolic dysfunction of the left ventricle. This condition is triggered by multiple emotional or physical stressors, while neurogenic stress cardiomyopathy after brain injuries has become increasingly recognized in children over the past few years. We report the case of an 11-year-old child with an atypical clinical presentation after a serious car crash accident. An initial computed tomography scan revealed an acute epidural hematoma, which was immediately treated by an emergency craniotomy. During the patient’s following pediatric intensive care unit hospitalization, severe hemodynamic instability was observed, leading to gradually higher doses of vasopressors for circulatory support. On echocardiography, the patient had signs of severe cardiac contractility compromise, with characteristic pattern of regional wall motion abnormalities of the left ventricle, which, in combination with seriously elevated cardiac enzymes, electrocardiographic (ECG) abnormalities and continuous thermodilution hemodynamic monitoring (PICCO) findings, led to intensification of inotropic support and to the diagnosis of takotsubo cardiomyopathy. Despite supportive measures, the patient developed multiorgan failure and succumbed to their serious illness. For this atypical case, extracorporeal membrane oxygenation (ECMO) was addressed as an option for the seriously failing heart, but due to the extremely high risk of intracranial bleeding, it could not be used for this patient’s treatment. In conclusion, Takotsubo cardiomyopathy should be suspected in pediatric cases of cardiac dysfunction after serious injuries or stress conditions.

## 1. Introduction

Patients with multiple traumatic injuries can be challenging to treat and are frequently complicated by multi-organ failure. Systemic inflammatory responses and heterogenous pathophysiological pathways, combined with the family’s or physicians’ emotional burden, compose a difficult puzzle [1]. Cardiogenic complications are uncommon, especially in pediatric patients, but need to be carefully sought out in critically injured patients.

Various causes of cardiogenic shock have been described in children. Congenital heart disease, cardiomyopathy and myocarditis are the most common, even though arrhythmias, cardiac surgery postoperative complications, endocarditis, rheumatic fever, Kawasaki disease, stress cardiomyopathy (takotsubo), valve cord rupture, drug or toxic substance intoxication and extracardiac diseases (sepsis-induced myocardial failure, pulmonary embolization, pneumothorax, tamponade) have also been associated with cardiogenic shock [2].

Takotsubo cardiomyopathy is an uncommon entity in children. The syndrome’s clinical presentation resembles that of acute myocardial infarction and consists of transient systolic dysfunction of the left ventricle, without evidence of coronary artery infarction. Takotsubo cardiomyopathy demonstrates typical wall motion abnormalities resembling that of a balloon, or of a traditional Japanese octopus fishing trap (“tako-tsubo”). The condition is triggered by major emotionally or physically stressful events, and typical clinical presentation includes chest pain, syncope and dyspnea. ST elevation is the classic electrocardiographic finding, and laboratory markers indicative of the condition should be sought, such as troponin and brain natriuretic peptide elevation [3]. A well-established relationship between traumatic brain lesions (especially subarachnoid hemorrhage) and neurogenic stress cardiomyopathy (takotsubo variant) has been described in adult patients. Several case reports have been published world-wide, pointing towards a similar correlation in pediatric brain injuries [4]. Takotsubo cardiomyopathy should be considered in the differential diagnosis of unexplained cardiac dysfunction in pediatric patients [5].

## 2. Case Presentation

In this case report, we demonstrate the devastating complications of an 11-year-old child with an atypical clinical presentation after a serious car–scooter accident. The initial physical examination at the Emergency Department revealed a Glasgow Coma Scale Score of 14/15 (opening their eyes on command), and no other pathological clinical findings. Past medical history was unremarkable, and no clinical risk factors had ever necessitated a cardiovascular evaluation in the past.

**Figure 1:**
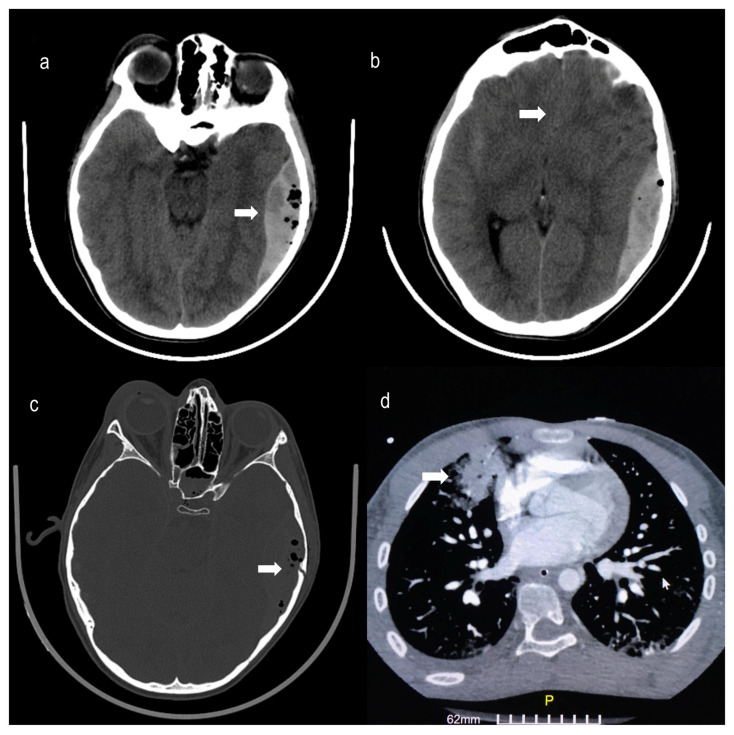
Brain Computed Tomography (CT) scan within 2 h of the accidentshowing: (**a**) acute left temporal and parietal epidural hematoma (arrow) (5 × 8 cm) with (**b**) mass effect and 4 mm midline shift (arrow), (**c**) Bone window of head CT scan showing a complex nondisplaced comminuted fracture of the left temporal bone (arrow), and (**d**) Chest CT showing contusions of the right upper lobe (arrow), right middle lobe and lower left lobe of the lungs (GE Healthcare Revolution 128-slice CT scanner, USA—Slice thickness of 1.25–5 mm with 3D Reconstructions).

Head computed tomography (CT) showed a complex nondisplaced comminuted fracture of the left temporal bone and a concomitant acute temporoparietal epidural hematoma (size of 2 × 5 × 8 (W × H × L) cm) presenting with mass effect, causing displacement of the third ventricle and 4 mm left to right midline shift. Radiologic signs of subarachnoid hemorrhage were also noted, and minor contusions were revealed in the temporoparietal area. Chest CT showed diffuse minor contusions of the lungs. Abdomen CT had no pathological findings (Figure 1).

An emergency craniotomy was performed to evacuate the epidural hematoma. During the surgical procedure, elevated levels of lactate (3.2 mmol/L) were noted, and hemodynamic instability ensued, necessitating high doses of vasopressors for circulatory support. Postoperatively, an intraparenchymal intracranial catheter was placed; initial intracranial pressure (ICP) values were 7–10 mmHg, and cerebral perfusion pressure was stable at 60–65 mmHg. Head CT scan showed complete evacuation of the hematoma and slight improvement of the midline shift.

**Figure 2:**
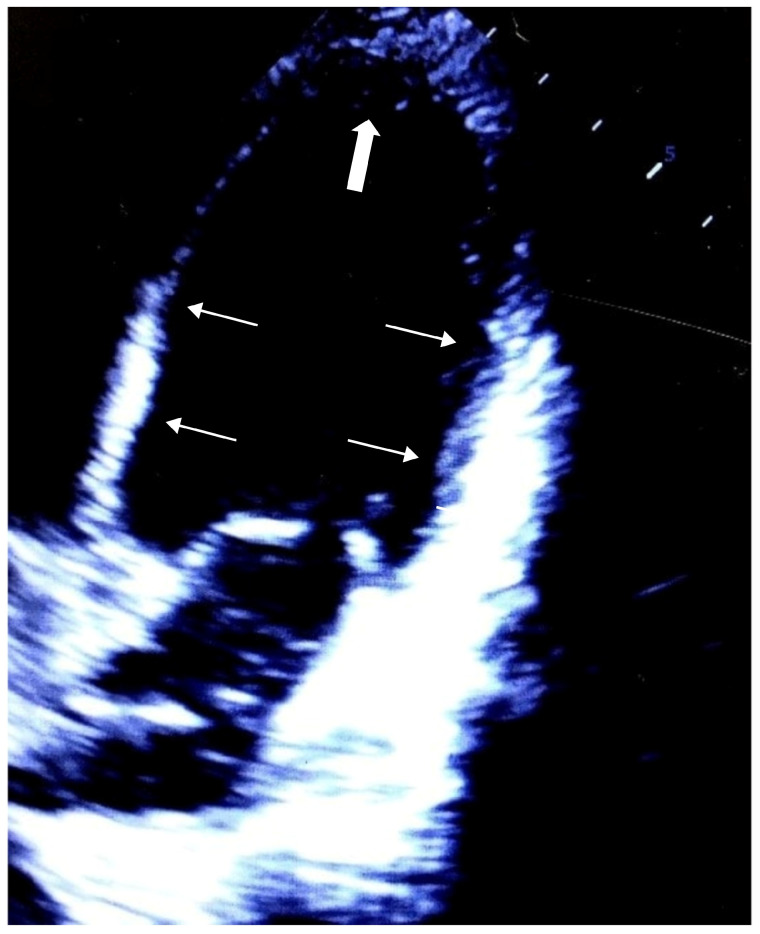
Echocardiography showing a severe compromise of cardiac contractility with regional wall motion abnormalities (thin arrows), apical hyperkinesia (thick arrow), along with reduced left ventricular ejection fraction (EF) of 40%, and fractional shortening (FS) of 28%. At end-systole the left ventricle resembles the basal ballooning pattern, giving the appearance of reverse Takotsubo cardiomyopathy (Video Recording of the Echocardiography: https://doi.org/10.6084/m9.figshare.20526903).

Upon their admission to the pediatric intensive care unit, the patient was placed on a controlled mode of ventilation, with stable, low levels of oxygenation support. However, a few hours after surgery, the patient developed further hemodynamic deterioration with a gallop rhythm and a gradually increasing need for norepinephrine infusion rates. The patient also suffered an episode of ventricular fibrillation, which spontaneously resolved with further increases in vasopressors. Despite vasopressor support, the patient continued to suffer from a profound circulatory shock. Echocardiography revealed a severe compromise of cardiac contractility with regional wall motion abnormalities, a reduced left ventricular ejection fraction of 40% and fractional shortening of 28% (Figure 2). Dynamic cardiac biomarkers (troponin 5000 mg/dL) and ECG alterations (ST segment elevations, repolarization abnormalities and abnormal Q-waves in V2–V6) were also noted. Continuous cardiac output monitoring by thermodilution/pulse contour analysis (PICCO system) also necessitated inotropic support intensification (cardiac index: 2–2.5 L/min/m^2^; global ejection fraction: 8%; systemic vascular resistance index: 1500 dyn × sec × cm^−5^ × m^2^), and after an ineffective trial of dobutamine, the patient was initially stabilized with the combination of milrinone and adrenaline continuous infusions. With the abovementioned initial clinical findings, the spectrum of diagnoses that could explain the acute cardiac failure was wide, including cardiac contusion, takotsubo cardiomyopathy or neurogenic stunned myocardium, spontaneous coronary dissection or even a viral myocarditis. However, the characteristic echocardiographic findings, with a regional hypokinetic pattern concerning mostly the midventricular and basal segments of the left ventricle, along with apical hyperkinesia, were significantly increasing the odds of reverse takotsubo cardiomyopathy.

For this atypical case, extracorporeal membrane oxygenation (ECMO) was addressed as an option for the seriously failing heart, but due to the extremely high risk of intracranial bleeding, this kind of support could not be considered for this patient’s treatment.

Within 48 h of the collision, and despite adequate sedation, the patient presented multiple episodes of intracranial hypertension (ICP values up to 30 mmHg), which were initially controlled with hyperosmotic therapy (iv bolus doses of NaCl 3% and/or mannitol).

After 60 h after the accident, the patient developed refractory cardiogenic shock and signs of multiorgan failure, despite maximal norepinephrine and vasopressin infusion rates. A brain computed tomography scan showed signs of cerebral oedema. Chest computed tomography scan depicted ground glass opacities, compatible with the onset of acute respiratory distress syndrome. Thoracic computed tomography angiography had no pathological findings.

Persistent intracranial hypertension (intracranial pressure 25–30 mmHg, cerebral perfusion pressure 45–50 mmHg), along with several episodes of bilateral mydriasis (pupil size of 8 mm, with no reaction to light), were soon noted, even though hyperosmolar therapy and barbiturate boluses were administered. The patient was arranged for an urgent decompressive craniectomy, but due to circulatory collapse, the patient soon succumbed to their serious illness. Necrotomy findings were compatible with massive pulmonary embolism due to severe decompensated heart failure, while myocardial tissue histology revealed no signs of myocarditis. Therefore, the diagnosis for this patient was mainly based on the characteristic echocardiographic takotsubo-like ventricular movement dysfunctions, which were related to severe traumatic brain injury and surgery as physical triggers. Based on these findings, takotsubo cardiomyopathy was considered as the most probable clinical scenario, since the patient met most of the proposed criteria by the Heart Failure Association from the European Society of Cardiology and the International Takotsubo Registry for this type of cardiomyopathy [6,7].

## 3. Discussion

Patients with multiple traumatic injuries pose significant challenges to clinicians and are often associated with devastating outcomes. In this article, we report the case of an 11-year-old child who suffered the serious cardiogenic complications of a severe brain injury. The management of the patient posed increased complexity both in terms of diagnosis and treatment decisions. Takotsubo cardiomyopathy secondary to severe brain injury was considered as the leading diagnosis, a rare occurrence in children, recently documented in 3.1 per 100.000 pediatric hospitalizations and with an overall mortality rate of 7% [2].

Takotsubo cardiomyopathy is generally defined in adults as a transient dyskinesia of the left ventricular apical segments with regional wall-motion abnormalities, extending beyond a single epicardial vascular distribution, with new electrocardiographic abnormalities, and is diagnosed after the exclusion of obstructive coronary disease, pheochromocytoma, myocarditis or hypertrophic cardiomyopathy [8].

The current postulated pathophysiology behind takotsubo cardiomyopathy suggests an emotional or physical stress-induced catecholamine surge, mediated in both the central and autonomic nervous systems, ultimately leading to direct microvascular dysfunction and inflammation, epicardial coronary vasoconstriction, increased cardiac workload and myocardial stunning. Although takotsubo cardiomyopathy is mostly diagnosed in post-menopausal women, predisposing factors for children and young adults are increasingly recognized nowadays, suggesting a similar pathophysiology for the pediatric population [8,9]. In children, however, atherosclerotic disease is rare, and takotsubo cardiomyopathy is more likely to be confused with an acute myocarditis syndrome or cardiac contusion after accidental injuries [10].

To the best of our knowledge, this is the first case report of reverse takotsubo cardiomyopathy in a pediatric patient, complicated by pulmonary embolism. Regional differences in myocardial adrenergic receptors could explain the typical contractility disorders of takotsubo cardiomyopathy, so a denser concentration of apex adrenoreceptors seems to account for the predominant apical involvement in most cases [11,12]. However, multiple variants of takotsubo cardiomyopathy have been reported, such as reverse takotsubo cardiomyopathy, which is a variant characterized by left ventricular basal akinesis/hypokinesis associated with apical hyperkinesia [13]. Mid-ventricular, global or even right ventricular contractility disorders have also been described in adults. Thus, the apical ballooning pattern is no longer considered pathognomonic [14].

Despite the fact that TC used to be considered a benign disease, recent data from international registries demonstrate complication rates as high as 35% [7]. However, in most of the published takotsubo cardiomyopathy cases for the pediatric population, early and remote prognosis are usually favorable. In a recent review in 17 pediatric patients with takotsubo cardiomyopathy, only two cases had persistent left ventricular systolic dysfunction, and in contrast to adults, takotsubo cardiomyopathy was found equally distributed between boys and girls in the pediatric age group. Moreover, only 60% of the cases had the classical apical ballooning pattern of dysfunction, whereas the proportion of non-apical takotsubo cardiomyopathy was found to be higher compared to adults [15].

Although differential diagnosis of takotsubo cardiomyopathy with myocarditis may also be challenging, history, clinical and laboratory details may help, since signs of infectious disease, higher levels of cardiac biomarkers or acute-phase proteins could point towards the diagnosis. Moreover, echocardiographic findings could also be preferable for identifying the reported takotsubo-like ventricular movement dysfunctions, which typically differ from the classic findings of cardiac contusion [16].

On the other hand, myocardial injury in blunt trauma mainly involves the right ventricle and atrium, compared with the less commonly involved left heart chambers. Finally, severe myocardial injury also carries a high mortality rate, especially when there is concomitant head injury, mostly at the site of the accident or during transportation [17,18]. Cardiac biomarkers have shown to be non-specific in the identification of myocardial injury, as they fail to discriminate between excessive or insignificant or between ischemic and non-ischemic cardiac injury [19]. Additionally, based on current literature, there are no standardized biomarkers that could lead to a definite diagnosis, especially for pediatric patients [20]. Regarding biomarkers, the degree of elevation of troponins, creatine phosphokinase (CPK) and creatine kinase-muscle/brain (CK-MB) does not indicate the amount of myocardial wall involvement [7].

To date, no randomized control trials have been conducted regarding the treatment for TC, and strategies for takotsubo cardiomyopathy are mainly supportive, while severe cases may require aggressive treatment options with mechanical left ventricular support [20,21]. Regarding extracorporeal membrane oxygenation, there is only one report in the literature with no hemorrhagic complications in a pediatric patient with stress-induced cardiomyopathy from acute brain hemorrhage, who was successfully decannulated after 57 h on extracorporeal membrane oxygenation [22]. In adults, several case series have challenged the notion that traumatic brain bleeding should be a contraindication to the use of extracorporeal membrane oxygenation. A number of patients have undergone this procedure without any new or recurring cerebral hemorrhage, even though the need for reduced coagulation has been disputed. However, increasing case reports in children reveal the need for establishing specific guidelines adjusted to the pediatric population.

Takotsubo syndrome has been related to many complications, such as arrhythmias, atrial fibrillation, thromboembolic events, mitral valve regurgitation or cardiac rupture [23]. The presented case’s post-mortem findings, which showed evidence of massive pulmonary embolism and coronary thrombi, posed the question of whether they represented typical complications of TC. The incidence of intraventricular thrombus and embolism in children has not been studied, but there are several systematic reviews in adult patients with a reported rate of 3.3% in takotsubo cardiomyopathy patients and a median interval of 2 days after disease onset [24]. The Virchow triad approach could presumably explain this, as a low blood state in the ventricle triggers thrombi formation. The coagulation cascade hypothesis has also been suggested as a possible mechanism, as endothelial markers and clotting activation biomarkers have been found to be elevated in these patients [23].

Post-traumatic coronary occlusion or dissection are also extremely rare in children, and their clinical presentation varies from completely asymptomatic to life-threatening conditions, such as arrhythmias, myocardial ischemia or cardiogenic shock. However, there is no preferred diagnostic modality, but computed tomography/magnetic resonance imaging or even cardiac catheterization have all been suggested. Cardiac catheterization is extremely disputable as a diagnostic tool in pediatric patients, as many side-effects have been reported in childhood, but there are some reports highlighting its curative value [25].

## 4. Conclusions

In conclusion, challenging cases with rare diagnoses demand awareness and a high-level of suspicion from the clinician that faces them. A multimodal and multidisciplinary approach with the assistance of a team of experts from multiple specialties and educational backgrounds is essential for the early recognition and treatment of these children. The lack of guidelines or expert-approved algorithms further complicates these cases, especially when controversial decisions arise or when contraindications limit the treatment options for these patients. The underfunding of several pediatric intensive care units seriously reduces the chances of survival for these critical patients that are not candidates for transport to specialized centers.

## Data Availability

The data used and/or analyzed during the current study are available from the corresponding author on reasonable request.
